# A Case of Human Oral Myiasis by *Lucilia sericata* in a Hospitalized Patient in Extremadura, Spain

**DOI:** 10.1155/2012/792683

**Published:** 2012-11-21

**Authors:** C. Pérez-Giraldo, I. Márquez-Laffón, M. T. Blanco, J. R. Muñoz del Rey, M. J. Chavero, M. A. Habela, A. C. Gómez-García

**Affiliations:** ^1^Microbiology Area, Department of Biomedical Sciences, Faculty of Medicine, University of Extremadura and CIBER-BBN, Avenida de Elvas s/n, 06007 Badajoz, Spain; ^2^Microbiology and Intensive Care Unit, Hospital Virgen del Puerto, 10600 Plasencia, Extremadura, Spain; ^3^Department of Parasitology, Faculty of Veterinary, University of Extremadura, Avenida de Elvas s/n, 06007 Badajoz, Spain

## Abstract

Myiasis is the term used to describe infestations, both obligatory and accidental, in vertebrate animals and humans by dipteral larvae. The oral cavity is rarely affected by this infestation and the circumstances which can lead to oral myiasis include persistent mouth opening together with poor hygiene, or facial traumatism. We present a case of oral myiasis by larvae of *Lucilia sericata*, a species present in the Iberian Peninsula, in a hospitalized patient with surgical problems.

## 1. Case History

A male aged 32 years was admitted to Intensive Care Unit (ICU) from General Surgery in a tertiary hospital (Hospital Virgen del Puerto, Plasencia, Extremadura, Spain). Case history revealed intestinal pathology with peritonitis, and laparotomy with intestinal resection, approximately a year previously. Two months before, the patient presented a new subocclusive condition, with gastroparesis, esophageal motor disorder, and intestinal myopathy. The patient was admitted to surgery with pain in the left iliac fossa compatible with acute peritonitis and perforation of the hollow viscus. An exploratory laparotomy was performed and the affected intestinal loops were resected. Two days later a further surgical intervention was necessary because of feverish syndrome and abdominal distension. During this period the patient remained under the effects of sedation. Abscesses and peritonitis with intestinal block were found, which prompted the patient's transfer to ICU. After 12 h in ICU a further intervention due to biliary peritonitis was required; the omentum was seen to be invaded by abscesses, requiring it to resected, a jejunostomy was performed, and the abdomen was left open. Antibiotherapy was administered contributing to improve the patient's evolution, with diminished fever and signs of inflammation. In the evolutionary period of 15 days following admission, the nurse reported mobile “maggots” in the patient's oral cavity, nasopharynx and oropharynx, and the presence of perilarval reaction. Treatment consisted of total mechanical removal of the larvae by combining manual extraction with tweezers and aspiration after having washed the infected area with water and povidone iodine. The patient's clinical condition was controlled without the appearance of further signs of infestation.

**Figure 1:**
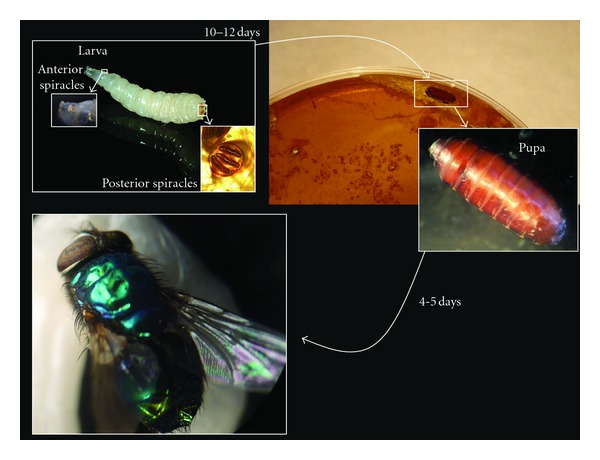
Aspect and evolution of the larvae of *Lucilia sericata* in our laboratory. Box picture of the posterior spiracles viewed under a microscope at low magnification.

Several fly larvae were sent to the laboratory, white in color, without legs, and with a smooth surface (Figure 1). The larvae presented 12 segments: the first of which had a pair of lateral papillae (anterior spiracles) with digitiform prolongations; the last segment having D-shaped posterior spiracles with 3 straight perimetral slots, not sinuous, and dark in color. The perimetral membrane is closed with a button which is especially evident when observed with low microscopic magnification (10x). These structures led us to identify this larva as part of the Calliphoridae family, belonging to the genus *Lucilia*. 

To confirm this diagnosis, a viable larva was incubated on an agar blood plaque (Figure 1) and kept in the laboratory at room temperature. The larva moved freely in the agar, and after about 10 days a dark brown structure appeared stuck on the edge of the plaque. This pupa was placed on a clean petri plate at room temperature. After a few days, the adult insect or imago appeared. Based on its subcostal sclerite, which was yellow in color, its meron structure, and the analysis of its wing venation, it was identified as *Lucilia sericata*, a species present in the Iberian Peninsula. 

## 2. Discussion

Insects such as dipterans may infest animals and humans, causing myiasis infection by fly larvae with multiple localizations [1, 2]. The larvae need to feed on live or dead tissues in order to grow and mutate. Although it is a well-known illness in the veterinary field, it is infrequent in man, being more frequent in the Tropics, with rare occurrences in warm zones [1].

Oral myiasis is a rare process in developed countries, but can occur anywhere [3]. It has been associated with poor oral hygiene [4, 5], open bite [6], neurological deficit or psychiatric disturbances [5, 7], alcoholism [8], and other disorders. In the episode described here the species involved was *L. sericata* which is distributed worldwide; this species is also known as the green bottle fly and sheep blowfly strike. Nosocomial myiasis, which occurs in a patient following hospitalization, is a very infrequent phenomenon [9]. Most reports of nosocomial myiasis have been of sporadic infestations, particularly in debilitated patients [10]. Factors contributing to nosocomial myasis include hypoesthesia or disturbed consciousness, which prevents the patient's sensation of fly contact [6], or paralysis that can prevent a patient from fending off a fly [11]. 

In this case, we believe that a sedated patient lying with his mouth half-open, in a rural hospital surrounded by pasturelands, could explain the entrance of flies into his oral cavity and the laying of eggs which hatched when the patient was in Intensive Care.

Myasis generates great anxiety, in both the patient and custodial personnel. The disease should be prevented by adequate control of insects, in addition to maintaining good oral and personal care of the patient, whatever their state of consciousness.
